# The Anthropometric Measure ‘A Body Shape Index’ May Predict the Risk of Osteoporosis in Middle-Aged and Older Korean People

**DOI:** 10.3390/ijerph19084926

**Published:** 2022-04-18

**Authors:** Bokun Kim, Gwon-min Kim, Eonho Kim, Joonsung Park, Tomonori Isobe, Yutaro Mori, Sechang Oh

**Affiliations:** 1 Department of Kinesiology, Silla University, Busan 46958, Korea; fabulousbobo79@gmail.com (B.K.); joon3750@silla.ac.kr (J.P.); 2 Department of Global Sports Coaching, In-je University, Gimhae 50834, Korea; 3 Department of Anti-ageing Health Care, Changwon National University, Changwon 51140, Korea; 4 Medical Research Institute, Pusan National University Hospital, Busan 49241, Korea; rlarnjsals47@gmail.com; 5 Department of Physical Education, Dongguk University, Seoul 04620, Korea; eonkim@dongguk.edu; 6 Faculty of Medicine, University of Tsukuba, Ibaraki 305-8575, Japan; tiso@md.tsukuba.ac.jp (T.I.); ymori@md.tsukuba.ac.jp (Y.M.); 7 Faculty of Rehabilitation, R Professional University of Rehabilitation, Ibaraki 300-0032, Japan

**Keywords:** a body shape index, obesity, osteoporosis, geriatrics

## Abstract

A body shape index (ABSI) is a recently introduced index of abdominal adiposity, relative to body mass index and height, and represents an alternative to body mass index and waist circumference. We aimed to determine whether ABSI is associated with osteoporosis and the ability of ABSI to predict osteoporosis, to investigate the relationship between obesity and osteoporosis In total, 6717 Korean participants (3151 men and 3566 women; 63.6 ± 8.5 years) were recruited and placed into the Normal, Osteopenia, or Osteoporosis groups on the basis of the minimum T-scores of the lumbar spine, proximal femur, and femoral neck. The T-scores of each region and ABSI were compared among the groups and odds ratios and cut-off values of ABSI for osteoporosis were calculated. In participants of both sexes, ABSI tended to increase as bone health deteriorated. The men and women in the highest quartile of ABSI were 1.887 and 2.808 times more likely to have osteoporosis, respectively, and the potential ABSI cut-off values for osteoporosis were 0.0813 and 0.0874 for male and female participants, respectively. These findings suggest that augmentation of ABSI and obesity is associated with a higher risk of osteoporosis and that ABSI may predict the risk of osteoporosis.

## 1. Introduction

Osteoporosis is a worldwide problem that is associated with adverse health outcomes and places a substantial burden on society [1,2,3]. Approximately two million people are diagnosed with an osteoporotic fracture each year worldwide, and the annual medical costs associated with the condition are predicted to reach approximately USD 25.3 billion in 2025 [3,4]. As the number of cases rise, and the quality of life of patients and the overall financial burden worsens, it is becoming increasingly important to identify individuals at high risk of osteoporosis and to institute management early, in order to reduce the prevalence of the condition.

Obesity has had significant impact on society and is closely related to a variety of chronic diseases [5,6,7,8,9,10,11,12]. However, the relationship between obesity and osteoporosis is controversial, despite numerous previous studies [13,14,15,16,17,18], possibly because of the inadequacy of existing anthropometric indexes. For decades, body mass index (BMI) has been the standard method of evaluating obesity, but it does not accurately estimate overall fat mass, nor does it discern visceral fat [19,20,21]. Waist circumference (WC) has been widely used in recent years to assess abdominal fat accumulation, which is associated with the onset of a number of disease conditions [22,23,24]. However, the utility of WC is also reduced by the influence of height [25]. Given these limitations of BMI and WC, it would be useful to identify another anthropometric index that is free of these limitations, in order to clarify the relationship between obesity and bone health.

A body shape index (ABSI) was presented in 2012. This is a method of quantifying abdominal adiposity relative to BMI and height that may represent a superior alternative to BMI or WC for the prediction of chronic disease [26]. The ABSI’s validity and reliability have been verified in various ethnicities of many countries [26,27,28,29], and ABSI has been reported to outperform BMI for the prediction of all-cause mortality [26]. In addition, because ABSI shows visceral adiposity regardless of BMI, it is also a potentially useful indicator of arterial stiffening [27]. Moreover, compared to BMI, it is a better indicator of the risks of diabetes and chronic kidney disease [28,29]. However, despite the utility of ABSI for the prediction of mortality and several chronic diseases, the relationship between ABSI and osteoporosis has not been properly explored [30,31,32].

We hypothesized that ABSI would predict the prevalence of osteoporosis, and aimed to investigate the relationship between obesity and osteoporosis, comparing its performance with those of WC and BMI. To this end, we performed a cross-sectional study of the relationship of ABSI with osteoporosis in middle-aged and older Korean people.

## 2. Materials and Methods

### 2.1. Study Design and Participants

**Figure 1:**
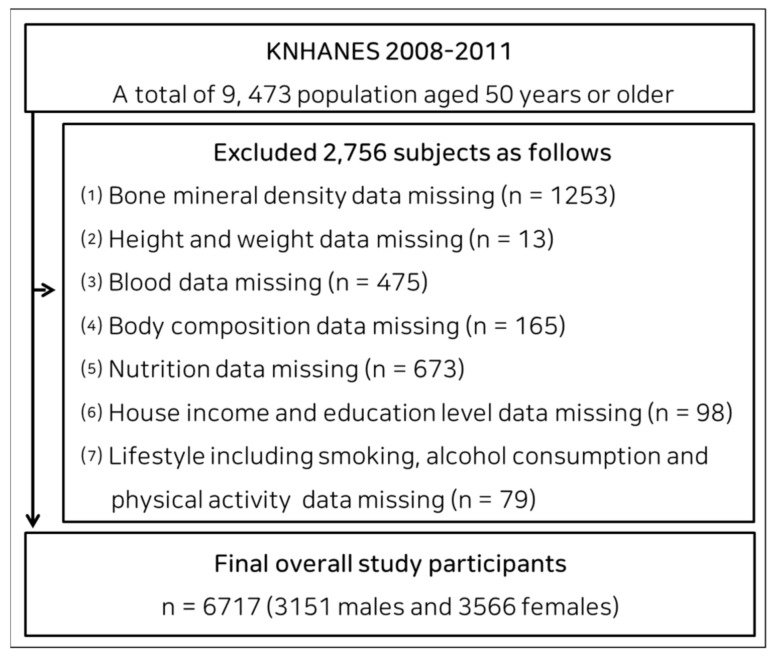
Flow chart of participants’ enrollment.

For this cross-sectional study, we extracted data from a database that was used to evaluate the general health, nutritional status, and lifestyle of South Korean people and that formed part of the Korea National Health and Nutrition Examination Survey (KNHANES), 2008–2011. Of the 9473 participants ≥50 years old in KNHANES 2008–2011, data from 6717 (3151 men and 3566 women) were included for analysis. A flow chart showing participant recruitment is presented in Figure 1.

Written informed consent was obtained from each participant, and the study was conducted following the principles of the Declaration of Helsinki and approved by the Institutional Review Board of the Korea Centers for Disease Control and Prevention approved KNHANES (2008-04EXP-01-C, 2009-01CON-03-2C, 2010-02CON-21-C, 2011-02CON-06-C).

### 2.2. Evaluation of Bone Health

The bone mineral density (BMD) (g/cm^2^) of the lumbar spine (L1–L4), proximal femur, and femoral neck were assessed utilizing dual-energy X-ray absorptiometry (DXA) (Discovery-W fan-beam densitometer; Hologic, Marlborough, MA, USA). Osteopenia and osteoporosis were diagnosed utilizing the minimum T-score of BMD at the three sites, depending on the criteria defined for KNHANES [33]. Concisely, the T-scores of the lumbar spine, proximal femur, and femoral neck were categorized as follows: ≥−1.0, normal; −2.49 to −1.01, osteopenia; and ≤−2.5, osteoporosis [33].

### 2.3. Anthropometric Indexes

Height was measured with a wall-mounted stadiometer, and WC was measured with a glass fiber tape to the nearest 0.1 cm. Body mass was measured to the nearest 0.1 kg using digital electronic scales (Jenix DS-102; Dong Sahn Jenix Co., Seoul, Korea). BMI was calculated as body mass (kg)/height squared (m^2^), and ABSI was calculated as WC/(BMI^2/3^ × height^1/2^) [26].

### 2.4. Statistical Analysis

Statistical analyses were performed utilizing SPSS software, version 20.0 (IBM, Inc., Armonk, NY, USA) [34,35]. Results are expressed as mean ± standard deviation (SD), with odds ratios (ORs) and a 95% confidence interval (CI). The independent *t*-test or the Mann–Whitney U test was used to make a comparison between men and women. One-way ANOVA was adopted to make a comparison of the three groups, followed by the Bonferroni post hoc test when appropriate (*p* < 0.05). For abnormally distributed data, the Mann–Whitney U test was adopted to compare the groups (*p* < 0.05). The Jonckheere–Terpstra test was used to compare the trends between the three groups (two-tailed, *p* < 0.05) [21,28]. Logistic regression was used to assess the relationship between ABSI and osteoporosis. The fully adjusted model was adjusted for the potential confounders age, educational and household income level, smoking status, medication use, alcohol consumption, moderate-to-vigorous physical activity, and nutritional factors that are suspected or known to affect osteoporosis. The optimal cut-off values of ABSI for the entire group, men, and women for the identification of osteoporosis were gathered with receiver operating characteristic (ROC) analysis using the area under the ROC curve (AUC), sensitivity, and specificity. This analysis was performed utilizing MedCalc for Windows ver. 9.1.0.1 (MedCalc^®^ Corp, Mariakerke Ostend, Belgium).

## 3. Results

The characteristics of the participants are displayed in Table 1. The average age of the overall, male and female participants was 63.6 (8.5), 63.1 (8.6), and 64.0 (8.4), respectively. There was a significant difference in ABSI between the sexes (*p* < 0.001), but not in WC or BMI. Extra characteristics of the participants are presented in the Supplementary Table S1.

Table 2 lists sex-specific differences and trends, depending on bone health category. Men accounted for 30.9%, 48.4%, and 20.7% of participants in the Normal, Osteopenia, and Osteoporosis groups, respectively, and women accounted for 23.7%, 49.2%, and 27.1%, respectively. The Osteopenia and Osteoporosis groups contained a higher ratio of women than men. There was a significant trend for T-score to decrease in the proximal femur, lumbar spine, and femur neck; and for height, body mass, WC, and BMI to decrease from the Normal to the Osteoporosis groups (SS: −46.51, −50.12, −49.50, −20.34, −23.38, −11.31, and −11.77, respectively; *p* < 0.001 for all); and the opposite trend was affirmed for age and ABSI (19.24 and 5.62, respectively; *p* < 0.001 for both). Post hoc testing demonstrated significant differences among the three groups with respect to all the parameters. The T-scores for the proximal femur, lumbar spine, and femoral neck; and the height, body mass, and WC decreased from the Normal to the Osteoporosis group, whereas age increased. The BMIs and ABSIs did not differ between the Osteopenia and Osteoporosis groups or between the Normal and Osteopenia groups. However, the values were significantly lower in the Osteoporosis group than in the Normal group. In women, there were significant trends for the T-score of the proximal femur, lumbar spine, and femoral neck to decrease, and for height, body mass, WC, and ABSI to decrease from the Normal to the Osteoporosis group (−47.95, −55.19, −51.51, −35.70, −36.32, −19.18, and −15.75, respectively; *p* < 0.001 for all); and the opposite trend was affirmed for ABSI (4.84, *p* < 0.001). There was no significant trend with respect to age. Post hoc testing cleared significant differences among the three groups with respect to all of the parameters. The T-scores for the proximal femur, lumbar spine, and femoral neck; and the height, body mass, WC, and BMI decreased from the Normal to the Osteoporosis group. The participants in the Osteopenia group were younger than those in the Normal and Osteoporosis groups. The ABSIs of the Osteopenia and Osteoporosis groups did not differ. However, they were higher than those of the Normal group. Table S2 shows the results of analyses of the entire group and comparisons of additional parameters in men and women.

**Figure 2:**
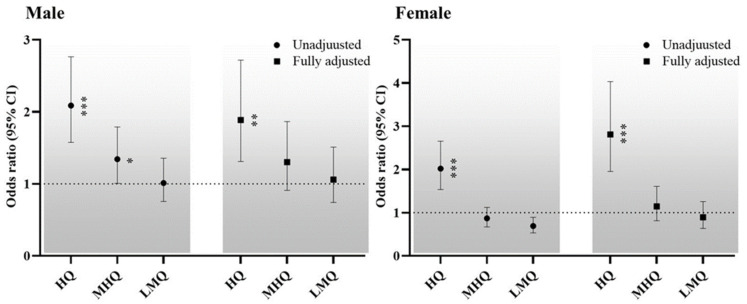
Sex-specific ORs for the relationship between ABSI and osteoporosis. Dotted line: reference; solid line: 95% confidence interval; Black circle: ORs. * *p* < 0.05, ** *p* < 0.01, *** *p* < 0.001 for the ORs for osteoporosis, compared with the lowest quartiles. Abbreviations: HQ, highest quartile; MHQ, middle-high quartile; LMQ, low-middle quartile.

The sex-specific ORs for the relationships between ABSI and osteoporosis are listed in Figure 2. The male and female participants were categorized according to quartile of ABSI into the lowest, low-middle, middle-high and highest quartiles. The unadjusted model cleared those men and women in the highest quartiles had ORs of 2.087 (95% CI: 1.576–2.764) and 2.019 (1.535–2.654), respectively, for osteoporosis, relative to the lowest quartiles. In addition, men in the middle-high quartile had an OR of 1.342 (1.007–1.789) for osteoporosis, compared to the lowest quartile. In the fully adjusted model, compared with the lowest quartiles, men and women in the highest quartiles had ORs of 1.887 (1.310–2.719) and 2.808 (1.954–4.034), respectively.

**Figure 3:**
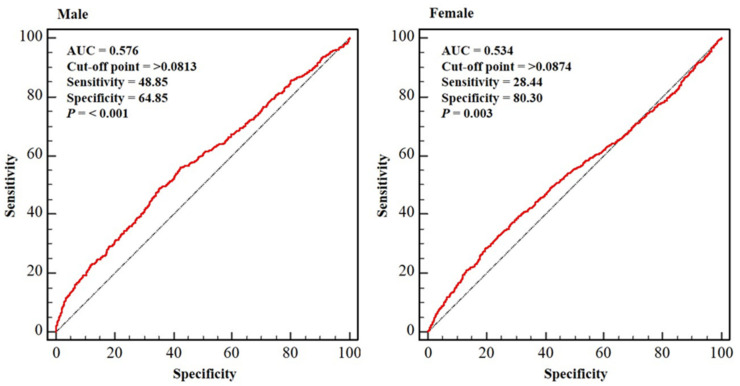
Sex-specific ROC curves indicating the ABSIs in the Osteoporosis group. Dotted blue line: reference; solid red line: area under the curve (AUC), indicating the accuracy of the A Body Shape Index (ABSI) for the identification of osteoporosis; cut-off value: the value of ABSI that predicts osteoporosis; sensitivity: the probability of individuals who actually have osteoporosis to be predicted to have osteoporosis; specificity: the probability of individuals who do not have osteoporosis to be predicted not to have osteoporosis. Abbreviation: AUC, area under curve.

Figure 3 presents the sex-specific ROC curves indicating the ABSIs in the Osteoporosis group. The optimal relationships between sensitivity and specificity were attained at ABSIs of 0.0813 and 0.0874 in male and female participants, respectively (*p* < 0.01 for each). For these cut-off values for osteoporosis, a sensitivity of 48.85% and a specificity of 64.85%; and a sensitivity of 28.44% and a specificity of 80.30% for male and female participants, respectively, were obtained.

## 4. Discussion

In the present cross-sectional study, we have identified an association between obesity and osteoporosis by assessing whether ABSI is associated with the prevalence of osteoporosis and analyzing the ability of ABSI to predict osteoporosis. The key findings were as follows. First, in both men and women, ABSI significantly increased as bone health deteriorated. Second, the male and female participants in the highest quartile of ABSI were 1.887 and 2.808, respectively, times more likely to have osteoporosis. Third, the ABSI cut-off values for osteoporosis were 0.0813 (sensitivity: 48.85% and specificity 64.85%) and 0.0874 (sensitivity: 28.44% and specificity: 80.30%) for male and female participants, respectively. There have been many previous studies on the relationship between osteoporosis and obesity, but the results have differed, owing to differences in the measurements used. However, the present findings suggest that obesity is positively associated with osteoporosis and increasing obesity, assessed using ABSI, is related to a higher prevalence of osteoporosis.

Osteoporosis is a condition in which bones lose their mass and strength as their density and quality diminish [36]. This can result in fracture, which induces pain and disability, and renders physical activity very difficult (17). For decades, it has been thought that the greater mechanical stimulation associated with the physical loading caused by obesity protects against osteoporosis [13,14,37]. However, the identification of leptin and other adipokines and the variety of molecular pathways through which they have their effects has provided mechanisms by which adipose tissue can communicate with bone [37,38]. These interactions include various elements, including pro-inflammatory cytokines, adiponectin, and leptin [37,38]. To fully evaluate the positive and negative effects of obesity, an anthropometric index is required that reflects abdominal fat mass, but also accounts for body mass, and thus physical loading, and height, which can reflect the size of the skeleton. Therefore, we aimed to clarify whether ABSI is related to osteoporosis and whether ABSI could be used to predict osteoporosis, in order to confirm an association between obesity and osteoporosis.

The present study revealed that the ABSI values for men in the Normal and Osteopenia groups were not different, but they were lower than those for the Osteoporosis group. For women, the ABSI values for the Osteopenia and Osteoporosis groups were not different, but were higher than that for the Normal group. There was a trend for ABSI to increase from the Normal to the Osteoporosis group in both sexes, and the highest quartiles had significant ORs (1.887 in men and 2.808 in women) relative to the lowest quartiles. Thus, a high ABSI is positively linked to the prevalence of osteoporosis in middle-aged and older Korean people. The present findings extend those of previous cross-sectional studies conducted in China and Turkey that identified positive associations between ABSI and osteoporosis [25,39]. Thus, it can be concluded that obesity has a negative influence on bone health.

**Figure 4:**
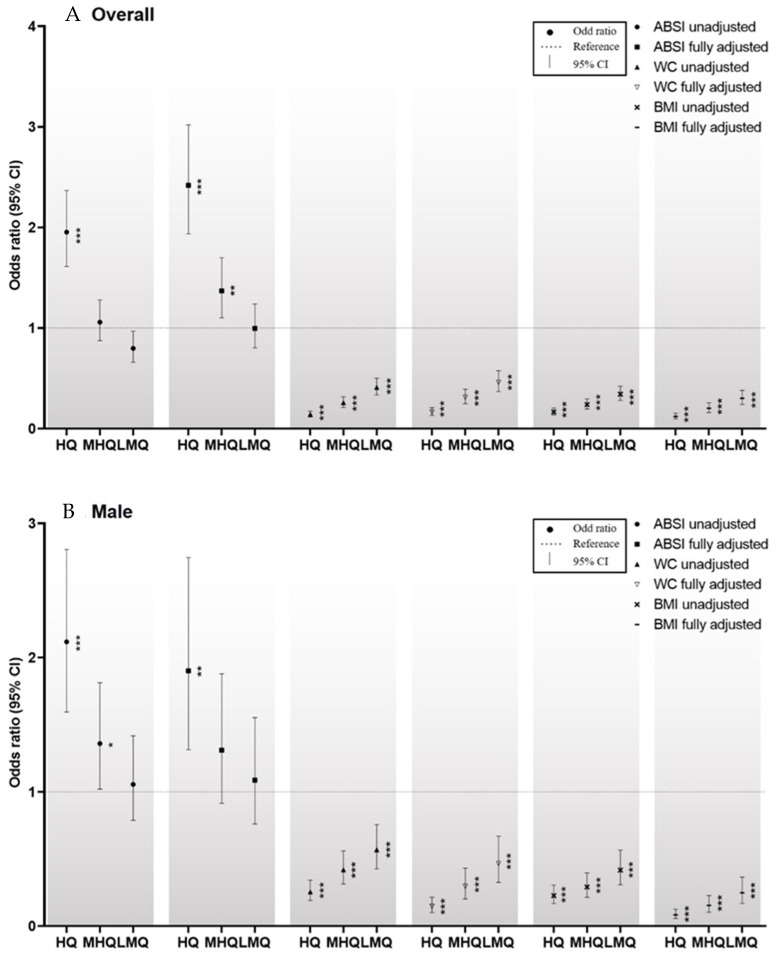

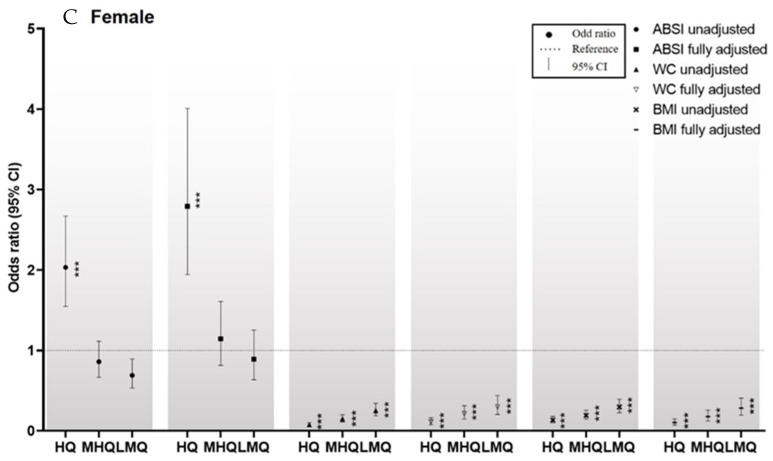
(**A**–**C**) Comparisons of the ORs for men, women, and the entire group for the relationships of the Z-scores of ABSI, WC, and BMI with osteoporosis. * *p* < 0.05, ** *p* < 0.01, and *** *p* < 0.001 for the ORs for osteoporosis, compared with the lowest quartile. Abbreviations: HQ, highest quartile; MHQ, middle-high quartile; LMQ, low-middle quartile. The ORs for the entire group for the relationships between the Z-scores for WC, BMI, and ABSI and osteoporosis are shown in Figure 4A. Depending on their Z-scores for WC, BMI, and ABSI, the participants were placed into quartiles. In the case of the Z-score for ABSI, in the unadjusted model, and compared to the lowest quartile, the highest quartile had an OR of 1.954 (95% CI: 1.613–2.367) for osteoporosis. In the fully adjusted model, and compared to the lowest quartile, the highest and middle-high quartiles had ORs of 2.202 (1.771–2.737) and 1.282 (1.036–1.585), respectively. As regards to the Z-score for WC, in the unadjusted model, and compared to the lowest quartile, the low-middle, middle-high, and highest quartiles showed ORs of 0.410 (0.335–0.503), 0.258 (0.211–0.317) and 0.142 (0.115–0.175), respectively for osteoporosis. In the fully adjusted model, and compared to the lowest quartile, the low-middle, middle-high, and highest quartiles had ORs of 0.461 (0.368–0.577), 0.313 (0.249–0.393), and 0.166 (0.131–0.210), respectively, for osteoporosis. With respect to the Z-score for BMI, in the unadjusted model, and compared to the lowest quartile, the low-middle, middle-high, and highest quartiles had ORs of 0.344 (0.280–0.421), 0.240 (0.195–0.294), and 0.166 (0.135–0.205), respectively, for osteoporosis. In the fully adjusted model, and compared to the lowest quartile, the low-middle, middle-high, and highest quartiles had ORs of 0.302 (0.240–0.380), 0.203 (0.160–0.256), and 0.120 (0.095–0.153), respectively, for osteoporosis.

In the present study, both WC and BMI were negatively correlated with the prevalence of osteoporosis (Table S2; Figure 4A–C), consistent with the findings of Sadiye and Gulsah (2021) [25]. However, Deng et al. (2020) [39] reported that the relationships of WC and BMI with osteoporosis are sex- and age-dependent. This result may have been obtained because of the confounding factors that affect WC and BMI as indexes of obesity. Taking the results of the present and previous studies together, ABSI appears to be superior to WC or BMI for assessing the relationship between obesity and bone health.

ROC curve analysis generated cut-off values of ABSI for osteoporosis of 0.0813 (sensitivity 48.85% and specificity 64.85%) and 0.0874 (sensitivity 28.44% and specificity 80.30%) for male and female participants, respectively. The relationship between ABSI and osteoporosis has been little investigated to date, and no previous study has generated cut-off values of ABSI for osteoporosis. Therefore, direct comparisons with other studies in this regard cannot be made. However, Nagaya et al. (2020) published an ABSI cut-off for systemic arterial stiffness of 0.080 for Japanese adults [40], Behboudi-Gandevani et al. (2016) pointed 0.077 and 0.078, respectively, as cut-offs for metabolic syndrome and insulin resistance, for young women [41]; and Kim et al. (2021) published cut-offs for chronic kidney disease of 0.0822 and 0.0795, respectively, in older men and women [29]. Taking the findings of the present and previous studies together, and taking into account age-, race-, and sex-related differences, an ABSI of 0.08 seems to be an appropriate threshold, above which metabolic derangement may occur, potentially leading to the onset of disease conditions.

There were both strengths and limitations to the present study. BMD was objectively assessed using DXA in a relatively large sample of middle-aged and older people. In addition, we controlled for significant potential confounders, including demographic factors and lifestyle, that may have influenced the relationship between ABSI and osteoporosis. However, it is uncertain whether the findings can be extrapolated to other ethnicities or countries. In particular, the AUC yielded by the ROC curve analysis was 0.576 and 0.534 in male and female participants, respectively. These values may indicate that the reliability of the cut-off value is not high enough. Therefore, further studies should be performed in individuals of other ethnicities to confirm the predictability of ABSI for osteoporosis.

## 5. Conclusions

We have confirmed that ABSI is positively associated with osteoporosis on the basis of the following findings. First, ABSI was found to increase as bone health deteriorated. Second, there was a higher prevalence of osteoporosis in the highest quartile of ABSI versus the lowest quartile. In addition, we have generated ABSI cut-off values for osteoporosis of 0.0813 and 0.0874 for men and women, respectively. Thus, the present findings suggest that obesity is positively associated with osteoporosis, which suggests that it is associated with a higher risk of osteoporosis. However, because studies investigating the association between ABSI and osteoporosis and assessing the predictability of ABSI for osteoporosis have involved only three different ethnicities in Korea, China, and Turkey, further studies involving other ethnic groups are essential to confirm the conclusions in the present study.

## Figures and Tables

**Table 1:** Characteristics of the study participants.

	Overall (n = 6717)	Male (n = 3151)	Female (n = 3566)	*p* Value
T score of total proximal femur BMD	−0.447 ± 1.026	−0.384 ± 1.028	−0.502 ± 1.021	<0.001
T score of lumbar spine BMD ^†^	−1.316 ± 1.368	−1.184 ± 1.335	−1.433 ± 1.386	<0.001
T score of femur neck BMD	−1.312 ± 1.085	−1.228 ± 1.065	−1.387 ± 1.098	<0.001
Age, year	63.6 ± 8.5	63.1 ± 8.6	64.0 ± 8.4	<0.001
Height, cm ^†^	159.3 ± 8.9	160.1 ± 8.5	158.6 ± 9.2	<0.001
Body mass, kg ^†^	60.9 ± 10.1	61.3 ± 9.8	60.5 ± 10.4	<0.01
Waist circumference, cm	83.8 ± 9.1	83.7 ± 9.1	83.8 ± 9.0	=0.653
Body mass index, kg/m^2^	24.0 ± 3.1	23.9 ± 3.1	24.0 ± 3.0	=0.276
A body shape index	0.0800 ± 0.0045	0.0799 ± 0.0044	0.0802 ± 0.0045	<0.05

Values are mean ± SD. ^†^ The Mann-Whitney U-test was used to assess differences between the groups.

**Table 2:** Differences between and trends across bone health groups in men and women.

	A. Normal (95% CI)	B. Osteopenia (95% CI)	C. Osteoporosis (95% CI)	*p* (Difference)	SS ^‡^	*p* (Trend ^‡^)
Males (N)	974	1524	653			
^T score^PF BMD ^†^	0.64 ± 0.67 (0.59, 0.68)	−0.53 ± 0.61 (−0.56, −0.50)	−1.56 ± 0.77 (−1.62, −1.50)	A > B > C	−46.51	<0.001
^T score^LS BMD ^†^	0.18 ± 0.91 (0.13, 0.24)	−1.37 ± 0.74 (−1.41, −1.33)	−2.79 ± 0.79 (−2.85, −2.73)	A > B > C	−50.12	<0.001
^T score^FN BMD ^†^	−0.13 ± 0.65 (−0.17, −0.09)	−1.38 ± 0.59 (−1.41, −1.35)	−2.51 ± 0.73 (−2.56, −2.45)	A > B > C	−49.50	<0.001
Age, year ^†^	59.6 ± 8.0 (59.1, 60.1)	63.4 ± 8.3 (63.0, 63.8)	67.7 ± 7.7 (67.1, 68.3)	A < B < C	19.24	<0.001
Height, cm ^†^	162.8 ± 7.9 (162.3, 163.3)	161.3 ± 7.7 (160.9, 161.6)	153.2 ± 7.5 (152.6, 153.8)	A > B > C	−20.34	<0.001
Body mass, kg ^†^	66.5 ± 9.6 (65.8, 67.1)	60.8 ± 8.5 (60.4, 61.2)	54.9 ± 8.5 (54.3, 55.6)	A > B > C	−23.38	<0.001
WC, cm ^†^	86.6 ± 8.7 (86.1, 87.2)	82.7 ± 8.7 (82.3, 83.2)	81.7 ± 9.5 (81.0, 82.5)	A > B > C	−11.31	<0.001
BMI, kg/m^2 †^	25.0 ± 2.9 (24.9, 25.2)	23.4 ± 3.0 (23.3, 23.6)	23.4 ± 3.3 (23.2, 23.7)	A > B, C	−11.77	<0.001
ABSI ^†^	0.0794 ± 0.0042 (0.0792, 0.0797)	0.0798 ± 0.0043 (0.0796, 0.0800)	0.0809 ± 0.0050 (0.0805, 0.0813)	A, B < C	5.62	<0.001
Female (N)	844	1755	967			
^T score^PF BMD ^†^	0.64 ± 0.69 (0.59, 0.69)	−0.50 ± 0.63 (−0.53, −0.47)	−1.50 ± 0.76 (−1.55, −1.45)	A > B > C	−47.95	<0.001
^T score^LS BMD ^†^	0.26 ± 0.94 (0.20, 0.33)	−1.42 ± 0.75 (−1.46, −1.39)	−2.93 ± 0.72 (−2.98, −2.89)	A > B > C	−55.19	<0.001
^T score^FN BMD ^†^	−0.04 ± 0.71 (−0.09, 0.01)	−1.44 ± 0.61 (−1.47, −1.41)	−2.47 ± 0.74 (−2.52, −2.43)	A > B > C	−51.51	<0.001
Age, year ^†^	67.5 ± 8.2 (67.0, 68.1)	60.6 ± 7.1 (60.3, 60.9)	67.0 ± 8.5 (66.4, 67.5)	A > B, B < C	0.11	=0.911
Height, cm ^†^	167.4 ± 6.2 (167.0, 167.8)	157.7 ± 7.9 (157.4, 158.1)	152.3 ± 7.4 (151.9, 152.8)	A > B > C	−35.70	<0.001
Body mass, kg	69.8 ± 8.6 (69.2, 70.3)	60.4 ± 8.5 (60.0, 60.8)	52.7 ± 8.0 (52.2, 53.2)	A > B > C	−36.32	<0.001
WC, cm ^†^	87.6 ± 7.4 (87.1, 88.1)	84.4 ± 8.8 (84.0, 84.8)	79.5 ± 8.9 (79.0, 80.1)	A > B > C	−19.18	<0.001
BMI, kg/m^2 †^	24.9 ± 2.6 (24.7, 25.0)	24.3 ± 3.0 (24.1, 24.4)	22.7 ± 3.0 (22.5, 22.9)	A > B > C	−15.75	<0.001
ABSI ^†^	0.0796 ± 0.0037 (0.0793, 0.0798)	0.0803 ± 0.0047 (0.0800, 0.0805)	0.0805 ± 0.0047 (0.0802, 0.0808)	A < B, C	4.84	<0.001

Values are mean ± SD. ^†^ The Mann-Whitney U test was used to compare groups. ^‡^ The Jonckheere-Terpstra test was used to assess the trends across the three groups. SS = standardized statistic; PF = proximal femur; LS = lumbar spine; FN = femoral neck; WC = waist circumference; BMI = body mass index; ABSI = A body shape index.

## Data Availability

The datasets analyzed during the current study are available from the corresponding author on reasonable request.

## Supplementary Materials

The following supporting information can be downloaded at: https://www.mdpi.com/article/10.3390/ijerph19084926/s1, Table S1. Characteristics and comparisons of study subjects. Table S2. The overall and gender-specific differences, and trends of subjects by bone health category.

## Abbreviations

**ABSI**: A Body Shape Index; **AUC**: Area Under the ROC Curve; **BMD**: Bone Mineral Density; **BMI**: Body Mass Index; **DXA**: Dual-Energy X-Ray Absorptiometry; **KNHANES**: Korea National Health and Nutritional Examination Survey; **ORs**: odds ratios; **ROC**: Receiver Operating Characteristic; **SD**: standard deviation; **SS**: standardized statistic; **WC**: Waist Circumstance.
