# Supplementary material for: The Anthropometric Measure ‘A Body Shape Index’ May Predict the Risk of Osteoporosis in Middle-Aged and Older Korean People
Source: Int J Environ Res Public Health. 2022 Apr 18;19(8):4926. doi: 10.3390/ijerph19084926 (PMC9026319; doi:10.3390/ijerph19084926)
Supplement: Supplementary file 1 [file ijerph-19-04926-s001.zip › ijerph-1607812-supplementary.pdf]

Supplementary Materials

Table S1. Characteristics of the study participants.

	Overall (n = 6717)	Male (n = 3151)	Female (n = 3566)	*P* Value
Lean mass, kg†	43.0 ± 8.7	43.4 ± 8.3	42.6 ± 9.0	< 0.001
Fat mass, kg†	17.4 ± 5.6	17.4 ± 5.7	17.4 ± 5.4	= 0.948
Percentage of fat mass, %†	28.8 ± 8.0	28.6 ± 8.2	29.1 ± 7.8	< 0.05
Systolic blood pressure, mm Hg	128.4 ± 17.7	127.3 ± 17.4	129.3 ± 17.9	< 0.001
Diastolic blood pressure, mm Hg†	78.7 ± 10.2	78.2 ± 9.9	79.1 ± 10.4	< 0.001
Glucose, mg/dL†	103.1 ± 26.0	102.4 ± 23.5	103.7 ± 28.0	= 0.984
Total cholesterol, mg/dL	194.6 ± 37.7	193.0 ± 37.8	195.9 ± 37.5	< 0.01
High density lipoprotein cholesterol, mg/dL	46.7 ± 11.3	46.9 ± 11.5	46.5 ± 11.1	= 0.154
Triglyceride, mg/dL	148.3 ± 08.2	145.7 ± 101.9	150.6 ± 113.5	= 0.064
Intake of energy, kcal/d	1810.9±746.4	1814.9 ± 737.4	1807.4 ± 754.3	= 0.684
Intake of carbohydrate, g	317.7±122.2	316.4 ± 119.6	318.9 ± 124.4	= 0.412
Intake of protein, g	61.7±33.3	62.0 ± 32.6	61.5 ± 33.9	= 0.539
Intake of fat, g	27.8±23.4	28.3 ± 23.5	27.3 ± 23.4	= 0.081
Medication (%)				
Hypertension	2404 (35.8)	1048 (33.3)	1356 (38.0)	< 0.001
Hyperlipidemia	589 (8.8)	201 (6.4)	388 (10.9)	< 0.001
Hyperglycemia	842 (12.5)	407 (12.9)	435 (12.2)	= 0.375
Moderate to vigorous physical activity (%)				
Low (< 150 min/week)	4219 (62.8)	1964 (62.3)	2255 (63.2)	= 0.443
High (≥ 150 min/week)	2498 (37.2)	1187 (37.7)	1311 (36.8)	
Houshold income (%)				
Low	2233 (33.2)	893 (28.3)	1340 (37.6)	< 0.001
Lower-middle	1724 (25.7)	808 (25.6)	916 (25.7)	
Upper middle	1367 (20.4)	695 (22.1)	672 (18.8)	
High	1393 (20.7)	755 (24.0)	638 (17.9)	
Education level (%)				
Primary school	3494 (52.0)	1114 (35.4)	2380 (66.7)	< 0.001
Middle school	1180 (17.6)	670 (21.3)	510 (14.3)	
High school	1343 (20.0)	816 (25.9)	527 (14.8)	
College	700 (10.4)	551 (17.5)	149 (4.2)	
Alcohol consumption (%)				
No drink for last a year	2764 (41.1)	1245 (39.5)	1519 (42.6)	< 0.05
≤ once a week	2507 (37.3)	1182 (37.5)	1325 (37.2)	
2-3 times/week	744 (11.1)	370 (11.7)	374 (10.5)	
≥ 4 times/week	702 (10.5)	354 (11.2)	348 (9.8)	
Smoking (%)				
Never	3859 (57.5)	1705 (54.1)	2154 (60.4)	< 0.001
Former smoking	790 (11.8)	413 (13.1)	377 (10.6)	
Current smoking	2067 (30.8)	1032 (32.8)	1035 (29.0)	

Values are mean ± SD. †The Mann-Whitney U test was used to compare the groups.

For the entire group and the male and female participants, the mean lean and percentage of fat masses were 43.0 (SD, 8.7), 43.4 (SD, 8.3), 42.6 (SD, 9.0), 28.8 (SD, 8.0), 28.6 (SD, 8.2), and 29.1 (SD, 7.8), respectively; and the mean systolic and diastolic blood pressures were 128.4 (SD, 17.7), 127.3 (SD, 17.4), 129.3 (SD, 17.9), 78.7 (SD, 10.2), 78.2 (SD, 9.9), and 79.1 (SD, 10.4), respectively. The mean total cholesterol concentrations were 194.6 (SD, 37.7), 193.0 (SD, 37.8), and 195.9 (SD, 37.5), respectively. With the exception of lean mass ($P < 0.001$), all the parameters, including the percentage fat mass ($P < 0.05$), systolic and diastolic blood pressure ($P < 0.001$ for both), and total cholesterol concentration ($P < 0.01$) were higher in women than men. Significant differences between the sexes were found with respect to hypertension, the use of anti-hyperlipidemic medication, household income, educational level, alcohol consumption, and smoking status ($P < 0.05$ for all). There were no sex differences for the other parameters.

Table S2. Comparisons between and trends across bone health groups in men, women, and the entire cohort

	A. normal (95% CI)	B. osteopenia (95% CI)	C. osteoporosis (95% CI)	*P* for difference	SS	*P* for trend‡
Overall						
Numbers	1818	3279	1620			
T score of TBF BMD†	0.64 ± 0.68 (0.61, 0.67)	-0.52 ± 0.62 (-0.54, -0.50)	-1.52 ± 0.76 (-1.56, -1.49)	A > B > C	-66.97	< 0.001
T score of LS BMD†	0.22 ± 0.93 (0.18, 0.26)	-1.40 ± 0.75 (-1.42, -1.37)	-2.87 ± 0.75 (-2.91, -2.84)	A > B > C	-74.81	< 0.001
T score of FN BMD†	-0.09 ± 0.68 (-0.12, -0.06)	-1.41 ± 0.60 (-1.43, -1.39)	-2.49 ± 0.74 (-2.52, -2.45)	A > B > C	-71.81	< 0.001
Age, year†	63.3 ± 9.0 (62.9, 63.7)	61.9 ± 7.8 (61.6, 62.2)	67.3 ± 8.2 (63.4, 63.8)	A > B, A < C, B < C	13.46	< 0.001
Height, cm†	164.9 ± 7.5 (164.6, 165.3)	159.4 ± 8.0 (159.1, 159.6)	152.7 ± 7.4 (152.3, 153.0)	A > B > C	-42.28	< 0.001
Body weight, kg†	68.0 ± 9.3 (67.6, 68.4)	60.6 ± 8.5 (60.3, 60.9)	53.6 ± 8.3 (53.2, 54.0)	A > B > C	-42.79	< 0.001
WC, cm†	87.1 ± 8.1 (86.7 87.5)	83.6 ± 8.8 (83.3, 83.9)	80.4 ± 9.2 (80.0, 80.9)	A > B > C	-21.40	< 0.001
BMI, kg/m^2†	25.0 ± 2.7 (24.8, 25.1)	23.9 ± 3.0 (23.8, 24.0)	23.0 ± 3.1 (22.8, 23.1)	A > B > C	-19.06	< 0.001
ABSI†	0.0795 ± 0.0039 (0.0793, 0.0797)	0.0800 ± 0.0045 (0.0799, 0.0802)	0.0807 ± 0.0048 (0.0804, 0.0809)	A < B < C	7.62	< 0.001
Z score of WC†	0.36 ± 0.90 (0.32, 0.40)	-0.02 ± 0.97 (-0.05, 0.01)	-0.37 ± 1.01 (-0.42, -0.32)	A > B > C	-21.40	< 0.001
Z score of BMI†	0.31 ± 0.89 (0.27, 0.35)	-0.04 ± 0.98 (-0.07, -0.01)	-0.33 ± 1.01 (-0.38, -0.28)	A > B > C	-19.06	< 0.001
Z score of ABSI†	-0.11 ± 0.88 (-0.15, -0.07)	0.01 ± 1.00 (-0.03, 0.04)	0.15 ± 1.07 (0.10, 0.20)	A < B < C	7.62	< 0.001
Lean mass, kg†	49.8 ± 8.1 (49.5, 50.2)	42.5±7.4 (42.3, 42.8)	36.2 ± 5.5 (36.0, 36.5)	A > B > C	-47.44	< 0.001
Fat mass, kg†	17.6 ± 5.4 (17.3, 17.8)	17.6 ± 5.7 (17.4, 17.8)	16.9 ± 5.5 (16.7, 17.2)	A, B > C	-2.11	< 0.05
Percentage of fat mass, %†	26.0 ± 7.2 (25.7, 26.4)	29.1 ± 8.2 (28.9, 29.4)	31.4 ± 7.4 (31.0, 31.7)	A < B < C	20.60	< 0.001
SBP, mm Hg	127.6 ± 17.5 (126.8, 128.4)	127.7 ± 17.5 (127.1, 128.3)	130.4 ± 17.9 (129.5, 131.3)	A, B < C	4.84	< 0.001
DBP, mm Hg†	80.3 ± 10.5 (79.8, 80.8)	78.5 ± 9.9 (78.2, 78.8)	77.2 ± 10.3 (76.7, 77.7)	A > B > C	-8.39	< 0.001
Glucose, mg/dL†	106.2 ± 28.5 (104.8, 107.5)	102.5 ± 25.7 (101.6, 103.4)	100.9 ± 23.3 (99.7, 102.0)	A > B > C	-7.54	< 0.001
Total Cholesterol, mg/dL	192.0 ± 37.3 (190.2, 193.7)	194.2 ± 38.0 (192.9, 195.5)	198.2 ± 37.1 (196.3, 200.0)	A, B < C	4.82	< 0.001
HDLC, mg/dL	45.69 ± 11.46 (45.2, 46.2)	47.00 ± 11.45 (46.6, 47.4)	47.21 ± 10.88 (46.7, 47.7)	A < B, C	4.99	< 0.001
Triglyceride, mg/dL†	158.3 ± 114.4 (153.0, 163.6)	147.2 ± 114.6 (143.3, 151.1)	139.2 ± 84.4 (135.1, 143.3)	A > B, C	-5.22	< 0.001
Intake of energy, kcal/d†	2095.5 ± 804.3 (2058.5, 2132.5)	1800.2 ± 721.6 (1775.5, 1824.9)	1513.3 ± 592.6 (1484.4, 1542.2)	A > B > C	-24.00	< 0.001
Intake of carbohydrate, g†	348.8 ± 125.5 (343.0, 354.6)	317.0 ± 122.3 (312.8, 321.2)	284.3 ± 108.3 (279.0, 289.5)	A > B > C	-16.36	< 0.001
Intake of protein, g†	73.6 ± 35.3 (71.9, 75.2)	61.5 ± 32.5 (60.4, 62.6)	48.9 ± 27.1 (47.5, 50.2)	A > B > C	-24.14	< 0.001
Intake of fat, g†	35.1 ± 25.9 (33.9, 36.3)	27.6 ± 23.5 (26.8, 28.4)	19.8 ± 17.0 (19.0, 20.7)	A > B > C	-23.21	< 0.001
Males						
Numbers	974	1524	653			
Z score of WC†	0.32 ± 0.96 (0.26, 0.38)	-0.11 ± 0.96 (-0.16, -0.06)	-0.21 ± 1.05 (-0.29, -0.13)	A > B > C	-11.31	< 0.001
Z score of BMI†	0.37 ± 0.92 (0.31, 0.42)	-0.16 ± 0.97 (-0.21, -0.11)	-0.16 ± 1.05 (-0.24, -0.08)	A > B, C	-11.77	< 0.001
Z score of ABSI†	-0.11 ± 0.95 (-0.17, -0.05)	-0.03 ± 0.98 (-0.08, 0.02)	0.22 ± 1.13 (0.14, 0.31)	A, B < C	5.62	< 0.001
Lean mass, kg†	47.3 ± 8.6 (46.7, 47.8)	43.8 ± 7.4 (43.4, 44.2)	36.8 ± 5.6 (36.3, 37.2)	A > B > C	-24.35	< 0.001
Fat mass, kg	18.6 ± 5.6 (18.3, 18.9)	16.5 ± 5.7 (16.2, 16.8)	17.7 ± 5.7 (17.3, 18.2)	A > B, A > C, B < C	-4.07	< 0.001

Percentage of fat mass, %†	28.3 ± 7.6 (27.8, 28.8)	27.3 ± 8.3 (26.8, 27.7)	32.1 ± 7.6 (31.5, 32.7)	A > B, A < C, B < C	7.83	< 0.001
SBP, mm Hg	127.2 ± 17.3 (126.1, 128.3)	125.2 ± 16.9 (124.3, 126.0)	132.4 ± 17.6 (131.1, 133.8)	A > B, A < C, B < C	4.60	< 0.001
DBP, mm Hg	79.5 ± 10.2 (78.8, 80.1)	77.7 ± 9.5 (77.2, 78.2)	77.4 ± 10.3 (76.6, 78.2)	A > B, C	-4.39	< 0.001
Glucose, mg/dL†	104.2 ± 25.1 (102.6, 105.8)	100.9 ± 21.8 (99.8, 102.0)	103.4 ± 24.5 (101.6, 105.3)	A > B, B < C	-1.72	= 0.085
Total Cholesterol, mg/dL	193.3 ± 38.1 (190.9, 195.7)	191.7 ± 37.2 (189.9, 193.6)	195.6 ± 38.4 (192.7, 198.6)	NS	0.86	= 0.388
HDLC, mg/dL	46.6 ± 12.0 (45.8, 47.3)	47.0 ± 11.5 (46.4, 47.6)	47.2 ± 11.0 (46.3, 48.0)	NS	1.89	= 0.059
Triglyceride, mg/dL	149.9 ± 102.2 (143.5, 156.4)	144.1 ± 106.3 (138.7, 149.4)	143.1 ± 90.4 (136.1, 150.1)	NS	-1.86	= 0.063
Intake of energy, kcal/d†	1981.5 ± 793.5 (1931.6, 2031.4)	1845.8 ± 721.5 (1809.6, 1882.1)	1494.1 ± 570.0 (1450.3 1537.9)	A > B > C	-12.48	< 0.001
Intake of carbohydrate, g	336.3 ± 121.5 (328.7, 344.0)	319.6 ± 121.0 (313.6, 325.7)	279.3 ± 104.1 (271.3, 287.3)	A > B > C	-9.20	< 0.001
Intake of protein, g†	68.6 ± 33.6 (66.4, 70.7)	63.5 ± 33.3 (61.8, 65.2)	48.7 ± 24.9 (46.8, 50.7)	A > B > C	-12.38	< 0.001
Intake of fat, g†	33.0 ± 25.7 (31.3, 34.6)	28.9 ± 23.5 (27.7, 30.1)	19.9 ± 16.7 (18.6, 21.2)	A > B > C	-12.73	< 0.001
Female						
Numbers	844	1755	967			
Z score of WC†	0.42 ± 0.82 (0.37, 0.48)	0.06 ± 0.98 (0.02, 0.11)	-0.47 ± 0.99 (-0.54, -0.41)	A > B > C	-19.18	< 0.001
Z score of BMI†	0.29 ± 0.87 (0.23, 0.35)	0.09 ± 1.00 (0.05, 0.14)	-0.43 ± 1.00 (-0.50, -0.37)	A > B > C	-15.75	< 0.001
Z score of ABSI†	-0.14 ± 0.81 (-0.19, -0.08)	0.01 ± 1.03 (-0.03, 0.06)	0.07 ± 1.06 (0.01, 0.14)	A < B, C	4.84	< 0.001
Lean mass, kg†	52.8 ± 6.4 (52.4, 53.2)	41.4 ± 7.3 (41.1, 41.8)	35.9 ± 5.4 (35.5, 36.2)	A > B > C	-40.59	< 0.001
Fat mass, kg†	16.3 ± 4.9 (16.0, 16.7)	18.5 ± 5.5 (18.2, 18.8)	16.4 ± 5.3 (16.0, 16.7)	A < B, B > C	0.43	= 0.668
Percentage of fat mass, %†	23.8 ± 6.0 (23.4, 24.2)	31.8 ± 8.3 (31.4, 32.2)	32.0 ± 7.9 (31.5, 32.5)	A < B, C	20.19	< 0.001
SBP, mm Hg	128.1 ± 17.8 (126.9, 129.3)	130.0 ± 17.8 (127.9, 130.2)	129.1 ± 18.0 (127.9, 130.2)	A < B	1.45	= 0.147
DBP, mm Hg	81.3 ± 10.7 (80.6, 82.0)	79.2 ± 10.1 (78.7, 79.7)	77.0 ± 10.3 (76.4, 77.7)	A > B > C	-8.00	< 0.001
Glucose, mg/dL†	108.4 ± 31.8 (106.3, 110.6)	103.9 ± 28.6 (102.6, 105.3)	99.2 ± 22.2 (97.8, 100.6)	A > B > C	-8.89	< 0.001
Total cholesterol, mg/dL†	190.4 ± 36.3 (188.0, 192.9)	196.4 ± 38.6 (194.6, 198.2)	199.9 ± 36.2 (197.6, 202.1)	A < B < C	5.26	< 0.001
HDLC, mg/dL†	44.7 ± 10.7 (44.0, 45.4)	47.0 ± 11.4 (46.5, 47.5)	47.2 ± 10.8 (46.5, 47.9)	A < B, C	5.23	< 0.001
Triglyceride, mg/dL†	167.9 ± 126.4 (159.4, 176.5)	149.9 ± 121.3 (144.3, 155.6)	136.6 ± 80.1 (131.6, 141.7)	A > B > C	-5.75	< 0.001
Intake of energy, kcal/d†	2227.0 ± 797.0 (2173.1, 2280.8)	1760.6 ± 719.5 (1726.9, 1794.3)	1526.3 ± 607.3 (1488.0, 1564.6)	A > B > C	-23.46	< 0.001
Intake of carbohydrate, g†	363.2 ± 128.6 (354.5, 371.9)	314.8 ± 123.4 (309.0, 320.6)	287.7 ± 110.9 (280.7, 294.7)	A > B > C	-13.76	< 0.001
Intake of protein, g†	79.4 ± 36.3 (76.9, 81.8)	59.8 ± 31.7 (58.4, 61.3)	48.9 ± 28.5 (47.1, 50.7)	A > B > C	-21.15	< 0.001
Intake of fat, g†	37.6 ± 25.9 (35.8, 39.3)	26.5 ± 23.4 (25.4, 27.6)	19.8 ± 17.2 (18.7, 20.9)	A > B > C	-19.53	< 0.001

Values are mean ± SD. †The Mann-Whitney U test was used to compare groups. ‡The Jonckheere-Terpstra test was used to assess trends across the three groups. SS = standardized statistic; PF = proximal femur; LS = lumbar spine; FN = femoral neck; WC = waist circumstance; BMI = body mass index; ABSI = a body shape index; SBP = systolic blood pressure; DBP = diastolic blood pressure; HDLC = high density lipoprotein cholesterol.

In the entire group, there were significant trends for the T-scores for the proximal femur, lumbar spine, and femoral neck; and the height, body mass, WC, BMI, lean and fat mass, DBP, glucose, triglyceride concentration, and energy, carbohydrate, protein, and fat intake to decrease from the Normal to the Osteoporosis group (SS: −66.97, −74.81, −71.81, −42.28, −42.79, −21.40, −19.06, −47.44, −2.11, −8.39, −7.54, −5.22, −24.00, −16.36, −24.14, and −23.21, respectively; $P < 0.05$ for all). There were also trends for age, ABSI, percentage fat mass, SBP, total cholesterol concentration, and HDLC concentration to increase (SS: 13.46, 7.62, 20.60, 4.84, 4.82, and 4.99, respectively; $P < 0.001$ for all). The Z-scores for WC, BMI, and ABSI showed the same trends as WC, BMI, and ABSI. *Post-hoc* testing demonstrated significant differences among the three groups with respect to all the parameters. The T-scores for the proximal femur, lumbar spine, and femoral neck, and height, body mass, waist circumstance, and BMI decreased from the Normal to the Osteoporosis group, whereas ABSI increased. The Z-scores for WC, BMI, and ABSI showed the same differences as WC, BMI, and ABSI. The participants in the Normal group were older than those in the Osteopenia group and younger than those in the Osteoporosis group. In addition, the participants in the Osteopenia group were younger than those in the Osteoporosis group. The Normal, Osteopenia, and Osteoporosis groups were ranked in descending order with respect to lean mass, DBP, glucose, and the

intakes of energy, carbohydrate, protein, and fat, but in ascending order for the percentage fat mass. Although the fat mass, SBP, and total cholesterol concentration of the Normal and Osteopenia groups did not differ, the fat mass, SBP, and total cholesterol concentration did significantly differ from those of the Osteoporosis group. The HDLC and triglyceride concentrations of the Osteopenia and Osteoporosis groups did not differ, but did differ from those of the Normal group.

In male participants, there were significant trends from the Normal to the Osteoporosis group for the Z-scores for WC and BMI, and lean and fat mass, DBP, and the intakes of energy, carbohydrate, protein, and fat to decrease (SS = −11.31, −11.77, −24.35, −4.07, −4.39, −12.48, −9.20, −12.38, and −12.73, respectively; $P < 0.001$ for all). The opposite trends were identified for the Z-score for ABSI, the percentage of fat mass and SBP (SS = 5.62, 7.83 and 4.60, respectively; $P < 0.001$ for both). There were no significant trends in glucose, total cholesterol, HDLC, or triglyceride concentration. *Post-hoc* testing showed significant differences among the three groups with respect to all the parameters, with the exception of the total cholesterol, HDLC, and triglyceride concentrations. The Normal, Osteopenia, and Osteoporosis groups ranked in descending order for the Z-score for WC, lean mass, and the intakes of energy, carbohydrate, protein, and fat. The fat mass of the Osteopenia group was lower than those of the Normal and Osteoporosis groups, and that of the Osteopenia group was lower than that of the Normal group. The percentage fat mass and SBP of the Normal group were higher and lower, respectively, than those of the Osteopenia and Osteoporosis groups, and those of the Osteopenia group were lower than those of the Osteoporosis group. The DBPs and Z-score for BMI of the Osteopenia and Osteoporosis groups did not differ, but were lower than that of the Normal group. The Z-score for ABSI of the Normal and Osteoporosis groups did not differ, but were lower than that of the Osteoporosis group. The glucose concentration of the Osteopenia group was lower than those of the Normal and Osteoporosis groups.

In female participants, there were significant trends from the Normal to the Osteoporosis group for the Z-scores for WC and BMI, and lean mass, DBP, glucose and triglyceride concentrations, and the intakes of energy, carbohydrate, protein, and fat to decrease (SS = −19.18, −15.75, −40.59, −8.00, −8.89, −5.75, −23.46, −13.76, −21.15, and −19.53, respectively; $P < 0.001$ for all). The opposite trend was found for the Z-score for ABSI, the percentage fat mass, and the total cholesterol, and HDL concentrations (SS = 4.84, 20.19, 5.26, and 5.23, respectively; $P < 0.001$ for all). However, there were no significant trends for fat mass or SBP. *Post-hoc* testing showed significant differences among the three groups with respect to all the parameters. The Normal, Osteopenia, and Osteoporosis groups ranked in descending order for the Z-scores for WC and BMI, and lean mass, DBP, glucose, triglyceride, and the intakes of energy, carbohydrate, protein, and fat; whereas total cholesterol concentration ranked in ascending order. The Z-scores for ABSI, and the percentage fat mass and HDLC concentrations of the Osteopenia and Osteoporosis groups did not differ, but were higher than those of the Normal group. The SBP of the Normal group was lower than that of the Osteoporosis group. The fat mass of the Osteoporosis group was higher than those of the Normal and Osteoporosis groups.
